# Upregulated in Hepatitis B virus-associated hepatocellular carcinoma cells, miR-331-3p promotes proliferation of hepatocellular carcinoma cells by targeting ING5

**DOI:** 10.18632/oncotarget.5642

**Published:** 2015-10-13

**Authors:** Yiyi Cao, Juan Chen, Dan Wang, Hong Peng, Xixi Tan, Dongmei Xiong, Ailong Huang, Hua Tang

**Affiliations:** ^1^ Key Laboratory of Molecular Biology on Infectious Diseases, Ministry of Education, Second Affiliated Hospital, Chongqing Medical University, Chongqing, China; ^2^ Collaborative Innovation Center for Diagnosis and Treatment of Infectious Diseases, Zhejiang University, Hangzhou, China

**Keywords:** HCC, HBV, miRNA, miR-331-3p, ING5

## Abstract

Hepatitis B virus (HBV) is a major risk factor for development and progression of hepatocellular carcinoma (HCC). It has been reported that viral infection can interfere with cellular microRNA (miRNA) expression and participate in the pathogenesis of oncogenicity. Our miRNAs array data indicated that miR-331-3p expression in HCC cell lines increased, but the relationship between miR-331-3p expression and HBV activity is unclear. Here, we observed elevated expression of miR-331-3p in different HCC cell lines expressing HBV. HBV, especially HBx, promotes miR-331-3p expression by enhancing its promoter activity. Using a miRNA target prediction database miRBase, we identified ING5 to be a novel target gene of miR-331-3p. miR-331-3p could inhibit ING5 expression by directly targeting its 3′-untranslated region (3′-UTR). As predicted, HBV was confirmed to repress ING5 at both mRNA and protein levels by promoting miR-331-3p expression. Our result indicated that miR-331-3p expression promotes proliferation of SMMC7721 cells by inhibiting ING5. ING5 overexpression promoted cell apoptosis in HCC cell lines. We also found ING5 expression was decreased in tumor tissue of HCC patient with HBV infection compared to its expression in para-carcinoma tissues. Conclusion: These results showed that miR-331-3p is upregulated by HBV and promotes proliferation of HCC cells though repression of ING5 expression. These data provide new insights for understanding the mechanisms of HBV-related HCC pathogenesis.

## INTRODUCTION

Hepatocellular carcinoma (HCC) is among the top three causes of cancer death in the Asian Pacific region [1]. Hepatitis B virus (HBV) of the hepadnavirus family confers major risk for HCC. In most high-risk HCC regions, HBV is associated with most cases of cirrhosis and at least 80% of HCC cases [2]. However, how HBV contributes to development of HCC is unclear.

MicroRNAs (miRNAs) are small single-stranded noncoding RNAs that regulate gene expression by interacting preferentially with the 3′untranslated regions (3′-UTRs) of target mRNAs, which may result in either inhibition of the target protein translation or degradation of the target mRNA [3, 4]. miRNAs and their target corresponding mRNAs form complex regulatory networks that are involved in cell proliferation, apoptosis, differentiation stress responses and other biological processes [5, 6]. miRNAs aberrant expression also contributes to a range of human pathologies, including cancer [7]. More and more reports have shown deregulation of miRNAs in human HCCs in recently years. Discovery of the critical role of miRNAs in modulating gene expression has not only changed our concept of gene expression regulation, but has also offered a new opportunity for designing anticancer strategies and therapies in HCC [8, 9].

As a major pathogen, did HBV involved in the dysregulation of miRNAs expression in HCC? It has been reported that HBV didn't encoded their own miRNAs but manipulate cellular miRNA expression [10]. miR-148a expression reduced growth, epithelial-to-mesenchymal transition, invasion, and metastasis of HBx-expressing HCC cells via inhibiting HPIP-mediated mTOR signaling [11]. miR-29c may contribute to tumor suppressive miRNA in the development and progression of HBV-related HCC by targeting TNFAIP3 [12]. miR-181 induces carcinogenesis in HBV-related HCC by targeting E2F5 and Fas [13, 14].

It has been reported that miR-331-3p promotes proliferation and metastasis of HCC by targeting the PH domain and leucine-rich repeat protein phosphatase (PHLPP) [15]. Recently, a miRNA microarray analysis showed that miR-331-3p was upregulated in a chronic HBV infection model HepG2.2.15 [16] cell line compared to the parental control HepG2 cell line [17]. Thus, the pathogenesis of miR-331-3p dysregulation in HBV-related HCC and the carcinogenesis of HBV and miR-331-3p in HCC development and progression warrants study.

To further study the function of miR-331-3p in HCC cell lines, we searched in the database for miR-331-3p candidate target genes, and detected gene expression in miR-331-3p over-expression SMMC7721 cells or HepG2 and HepG2.2.15 cells by using qRT-RCR. ING5 can be downregulated by miR-331-3p and also inhibited by HBV, so we chosen ING5 for our next step research. The inhibitor of growth 5 (ING5) gene encodes a 28-kD protein [18] and its over-expression in colorectal cancer cells diminished colony-forming efficiency, decreased the number of cells in the S phase, and induced apoptosis in a p53-dependent manner [19]. Altered ING5 expressions have been reported in certain cancers including gastric carcinogenesis, human head and neck squamous cell carcinoma, and oral squamous cell carcinoma, functioned as a suppressor by inhibiting cell growth and inducing apoptosis [20–22]. However, as a tumor suppressor gene, ING5 functions have not been reported in HCC. The discovery of HBV, miRNAs, and target gene interactions may offer insight into mechanisms of HCC as well as provide novel therapeutic strategies for treating HCC.

## RESULTS

### HBV upregulates expression of miR-331-3p by enhancing its promoter activity

**Figure 1:**
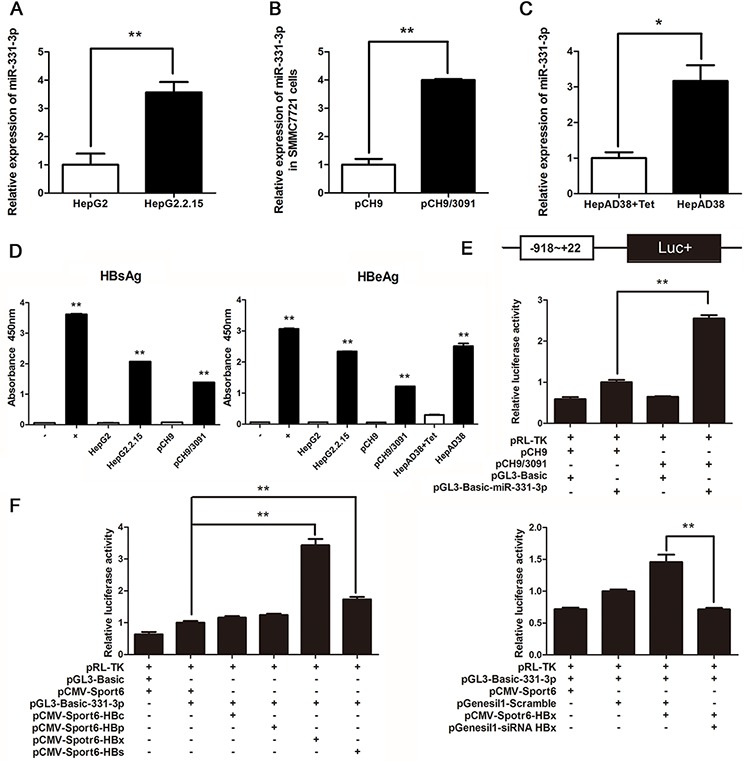
HBV upregulates miR-331-3p expression by enhancing its promoter activity **A–C.** Relative miR-331-3p expression in HepG2 and HepG2.2.15 cells (A), SMMC7721 cells transfected with pCH9 or pCH9/3091 plasmids (B) and HepAD38 cells with/without Tet treatment (C) miRNA was normalized to U6 RNA. **D.** HBsAg and HBeAg expression in HCC cell lines. **E.** miR-331-3p promoter activity in SMMC7721 cells transfected with pCH9 or pCH9/3091 measured by Dual luciferase reporter analysis. **F.** miR-331-3p promoter activity in SMMC7721 cells co-transfected with DNAs or RNAs as indicated and measured by Dual luciferase reporter analysis. *P < 0.05, **P < 0.01.

Firstly, expression of miR-331-3p in a panel of cell lines including HepG2, HepG2.2.15 was measured using qRT-PCR. miR-331-3p expression was greater in HepG2.2.15 cells compared to HepG2 cells (Figure 1A), that consistent with previous miRNAs microarray data [17]. In a similar approach, another HCC cell line SMMC7721 cells were transfected with pCH9 (vector control) or pCH9/3091 (HBV-expressing) plasmids, and their miR-331-3p expression was measured. Again, miR-331-3p expression was increased in SMMC7721 transiently expressing HBV compared to controls (Figure 1B). miR-331-3p expression was also suppressed in tetracycline (Tet) treated HepAD38 compared to those in untreated cells (Figure 1C). HBsAg and HBeAg were measured by ELISA in three cell lines to confirm HBV expression (Figure 1D). Therefore, HBV promoted miR-331-3p expression in different HCC cell lines.

To investigate the mechanism of miR-331-3p upregulation by HBV, we constructed pGL3-Basic-miR-331-3p vector which contains the miR-331-3p promoter for the luciferase reporter according to luciferase reporter assay. miR-331-3p promoter activity was higher in the pCH9/3091 and pGL3-Basic-miR-331-3p co-transfected group compared to pCH9 and pGL3-Basic-miR-331-3p co-transfected group (Figure 1E).

To investigate the possible mechanism by which HBV regulate miR-331-3p promoter activity, viral proteins HBc, HBp, HBx and HBs expression plasmids [23] were transfected respectively in HCC cells expressing miR-331-3p. Dual luciferase reporter assay found that viral protein HBx significantly enhanced miR-331-3p promoter activity while HBx knockdown inhibited its promoter activity (Figure 1F). These data suggested that HBV, especially HBx, could upregulate miR-331-3p expression by increasing its promoter activity.

### miR-331-3p promotes proliferation of HCC cells

**Figure 2:**
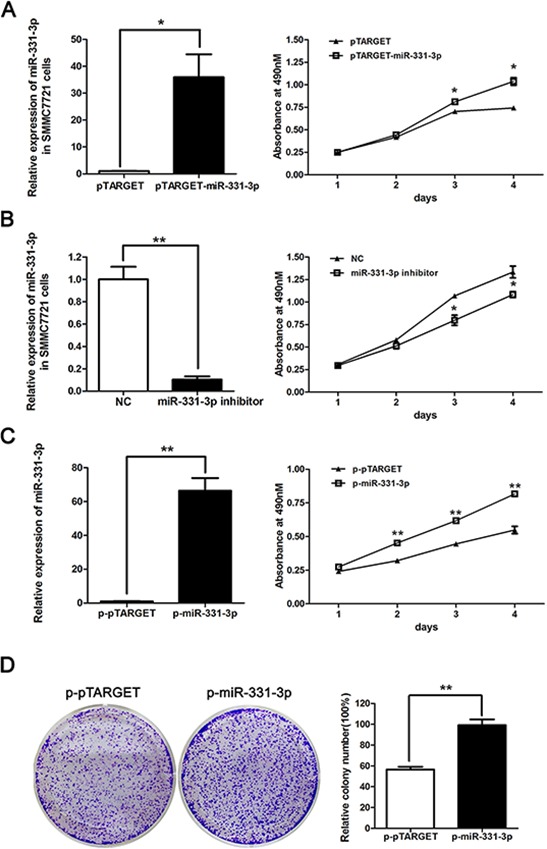
miR-331-3p promotes proliferation of HCC cells **A.** Relative miR-331-3p expression in pTARGET-miR-331-3p and pTARGET transfected SMMC7721 cells and effect of miR-331-3p over-expression on cell proliferation measured by MTS assay. **B.** Relative miR-331-3p expression in miR-331-3p inhibitor or NC transfected SMMC7721 cells and effect of miR-331-3p inhibitor on cell proliferation measured by MTS assay. **C.** Relative miR-331-3p expression in p-miR-331-3p compares to p-pTARGET stable cell lines, p-miR-331-3p and p-pTARGET cell proliferation were measured by MTS assay. miRNA was normalized to U6 RNA. **D.** Representative pictures of colony formation assay with p-pTARGET and p-miR-331-3p. Colonies were counted and values are reported as ratios. *P < 0.05, **P < 0.01.

After we confirmed a correlation between miR-331-3p and HBV expression, we functionally characterized miR-331-3p by studying its effect on HCC cell proliferation. SMMC7721 cells were transiently transfected with pTARGET, pTARGET-miR-331-3p, inhibitor NC or miR-331-3p inhibitor, and miR-331-3p relative expression was measured by qRT-PCR. Cell proliferation was measured using MTS assay. miR-331-3p expression was greater in pTARGET-miR-331-3p transfected SMMC7721 cells compared to the vector pTARGET transfected cells. As shown in Figure 2A, miR-331-3p overexpression significantly promoted cell proliferation. When SMMC7721 cells were transfected miR-331-3p inhibitor, miR-331-3p expression was lower and cell proliferation was repressed compared to those in NC cells (Figure 2B).

To confirm these data, SMMC7721 cell lines stably expressing miR-331-3p, p-miR-331-3p and its negative control p-pTARGET, were constructed. Overexpression of miR331-3p was confirmed using qRT-PCR. MTS and colony formation assay results indicated that both cell and colony numbers were increased in p-miR-331-3p-expressing cells compared to control cells (Figure 2C and 2D). Therefore, miR-331-3p can promote cell proliferation in HCC cell lines.

### ING5 is a target gene of miR-331-3p

**Figure 3:**
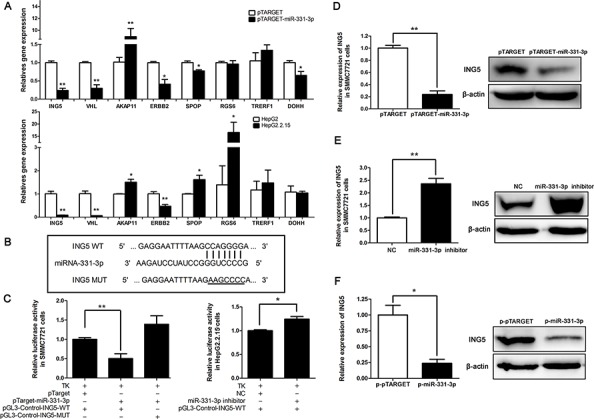
ING5 is a target gene of miR-331-3p **A.** Relative gene expression in pCH9 and pCH9/3091 transfected SMMC7721 cells (upper panel) and in HepG2 and HepG2.2.15 cells (lower panel). **B.** Schematic diagram of predicted miR-331-3p binding site WT or MUT in the ING5 3′-UTR. **C.** Luciferase reporter assays in SMMC7721 (left panel) or HepG2.2.15 cells (right panel), co-transfected with DNAs or RNAs as indicated. **D.** ING5 mRNA and protein expression in SMMC7721 cells transfected with pTARGET-miR-331-3p or its control. **E.** ING5 mRNA and protein expression in SMMC7721 cells transfected with miR-331-3p inhibitor or NC. **F.** ING5 mRNA and protein expression in p-pTARGET and p-miR-331-3p. β-actin was used as an internal quantitative control. *P < 0.05, **P < 0.01.

To better understand mechanisms of growth regulation by miR-331-3p, a bioinformatic website miRBase (http://mirbase.org/index.shtml) was used to search for downstream genes of miR-331-3p. Using website data, we screened several target genes and compared their expression in pTARGET or pTARGET-miR-331-3p transfected SMMC7721 cells. We found that ING5, VHL, ERBB2 and SPOP expression were inhibited by miR-331-3p overexpression. DOHH, a gene that has been reported to be a target gene of miR-331-3p [25] was included as a positive control. We then examined expression of these genes in HepG2 and HepG2.2.15 cells. Again ING5, VHL and ERBB2 expression were suppressed in the HBV-expressing HepG2.2.15 cells that have elevated expression of miR-331-3p (Figure 3A). We finally chose ING5 for subsequent studies.

MiRNA regulates gene expression by interacting preferentially with the 3′-untranslated regions (3′-UTRs) of its target mRNAs. To confirm that ING5 is a target gene of miR-331-3p, pGL3-Control-ING5-WT containing the 3′-UTR binding site (CCAGGGG) for miR-331-3p, and the pGL3-Control-ING5-MUT containing a mutated site (AAGCCCC) was constructed (Figure 3B). The 3′-UTR binding site was predicted in miRDB. A luciferase assay showed that luciferase activity was lower in SMMC7721 cells co-transfected with pTARGET-miR-331-3p and pGL3-Control-ING5-WT than that in SMMC7721 cells co-transfected with pTARGET and pGL3-Control-ING5-WT, and recovered in SMMC7721 cells co-transfected with pTARGET-miR-331-3p and pGL3-Control-ING5-MUT. When HepG2.2.15 cells were co-transfected with miR-331-3p inhibitor and pGL3-Control-ING5-WT, luciferase activity was increased (Figure 3C). These data suggested that miR-331-3p significantly decreased luciferase activity of the ING5 WT 3′-UTR but not the mutant 3′-UTR.

Next, qRT-PCR and Western blot analyses were used to assess effects of miR-331-3p on ING5 expression in HCC cells. Our data showed that ING5 expression decreased in SMMC7721 cells overexpressing miR-331-3p at both mRNA and protein levels (Figure 3D). On the other hand, when miR-331-3p expression was inhibited we observed an increase of ING5 expression in the SMMC7721 cells (Figure 3E). This also occurred in p-pTARGET and p-miR-331-3p cells (Figure 3F). Taken together, we conclude that ING5 is a target gene of miR-331-3p and ING5 expression is inhibited by miR-331-3p in HCC cell lines.

### HBV represses ING5 expression by upregulating miR-331-3p

**Figure 4:**
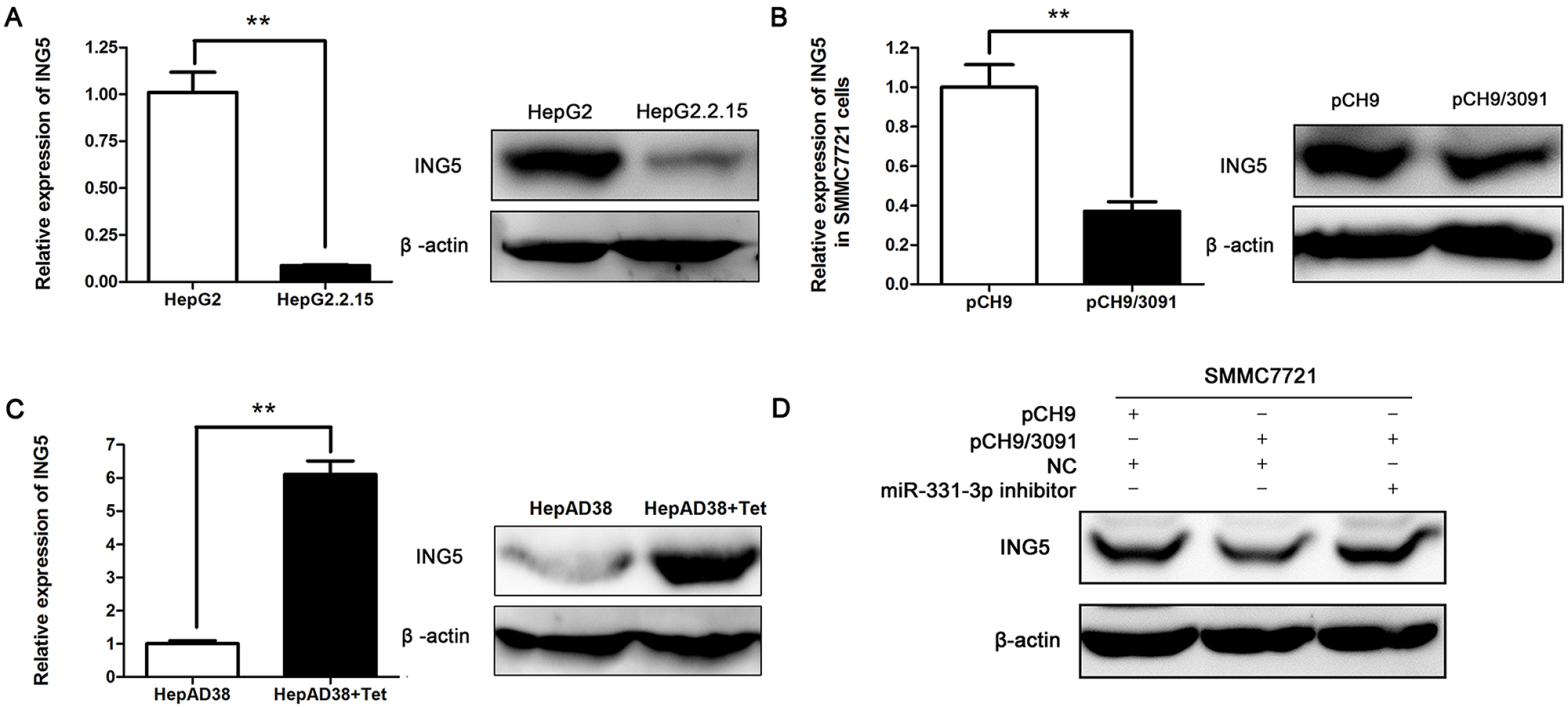
HBV represses ING5 expression by upregulating miR-331-3p **A–C.** ING5 mRNA and protein expression in HepG2 and HepG2.2.15 cells (A), pCH9 and pCH9/3091 transfected SMMC7721 cells (B) and HepAD38 cells treated with Tet or not (C) β-actin was used as an internal quantitative control. *P < 0.05, **P < 0.01. **D.** ING5 protein expression in SMMC7721 cells cotransfected with DNAs or RNAs as indicated.

We next investigated the regulating effect between HBV and ING5. At first, we examined ING5 expression in HBV-expressing HCC cell lines. Both qRT-PCR and Western blot results showed that ING5 expression was inhibited in HepG2.2.15 and HBV transiently transfected SMMC7721 cells (Figure 4A and 4B). Upregulated ING5 expression was also observed in Tet treated HepAD38 cells compared to untreated cells (Figure 4C). We concluded that ING5 expression could be inhibited by HBV.

We next assessed whether HBV inhibited ING5 expression by regulating miR-331-3p. To this end, SMMC7721 cells were co-transfected with pCH9/3091 and miR-331-3p inhibitor. As shown in Figure 4D, ING5 protein expression was reduced in SMMC7721 cells expressing only HBV (co-transfection with NC and pCH9/3091) and reversed when the cells express both HBV and miR-331-3p inhibitor (co-transfection with miR-331-3p inhibitor and pCH9/3091). This result suggested that HBV represses ING5 expression by upregulating miR-331-3p.

### ING5 inhibits HCC cell proliferation

**Figure 5:**
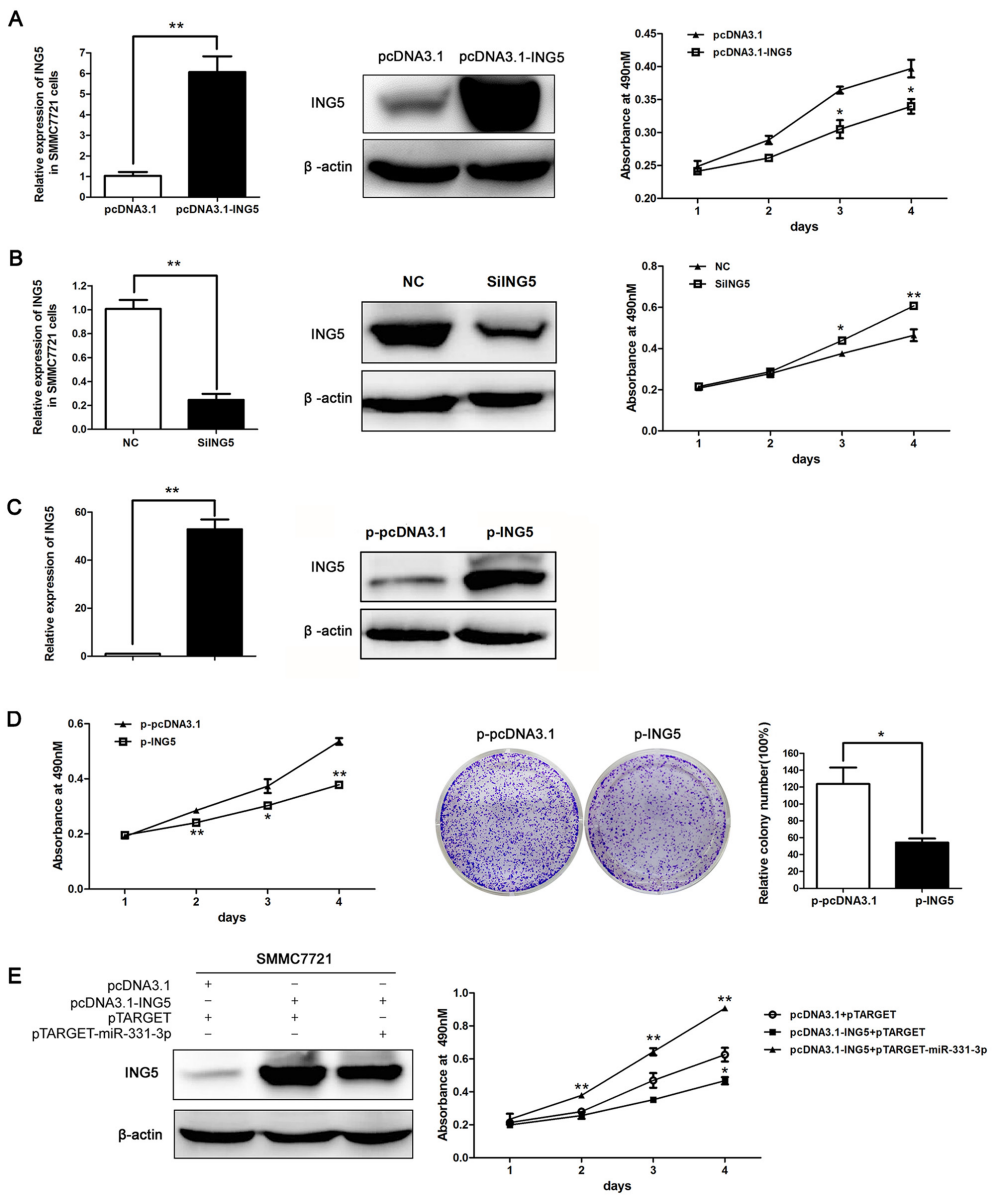
ING5 inhibited HCC cell proliferation **A.** ING5 mRNA and protein expression in SMMC7721 cells transfected with pcDNA3.1 or pcDNA3.1-ING5, effect of ING5 overexpression on cell proliferation was measured by MTS assay. **B.** ING5 mRNA and protein expression in siING5 transfected SMMC7721 cells compared to NC; effect of ING5 silencing on cell proliferation was measured by MTS assay. **C.** ING5 mRNA and protein expression in p-pcDNA3.1 and p-ING5 stable cell lines. (A-C) β-actin was used as an internal quantitative control. **D.** p-ING5 and p-pcDNA3.1 cell proliferation measured by MTS and colony formation assays. Colonies were counted and values are reported as ratios. **E.** ING5 protein expression in SMMC7721 cells co-transfected with DNAs as indicated. MTS assay was used to measure cell proliferation of SMMC7721 cells co-transfected with DNAs as indicated. *P < 0.05, **P < 0.01.

To assess how ING5 modulates HCC cell proliferation, we constructed a pcDNA3.1-ING5 vector that could over express ING5 (the 3′-UTR sequence of ING5 was not included) and an ING5 siRNA. As expected, ING5 over-expression inhibited cell proliferation and ING5 silencing promoted cell proliferation (Figure 5A and 5B).

Then, we constructed an ING5 stably expressing cell line p-ING5 and a control group p-pcDNA3.1 based on SMMC7721. Over-expression efficiency was measured with qRT-PCR and Western blot (Figure 5C). MTS and colony formation assay data indicated that cell proliferation and number of colonies decreased in p-ING5 compared to p-pcDNA3.1 (Figure 5D). Thus, ING5 can repress HCC cell proliferation.

Finally, we transfected SMMC7721 with three vector groups as indicated in Figure 5E. ING5 expression was increased in pcDNA3.1-ING5 and pTARGET co-transfected groups and decreased in pcDNA3.1-ING5 and pTARGET-miR-331-3p co-transfected groups as measured by Western blot analysis. MTS assay indicated that cell proliferation was decreased by ING5 and increased by miR-331-3p (Figure 5E). Therefore, miR-331-3p promotes cell proliferation at least partly by inhibiting ING5.

### ING5 induces HCC cell apoptosis

**Figure 6:**
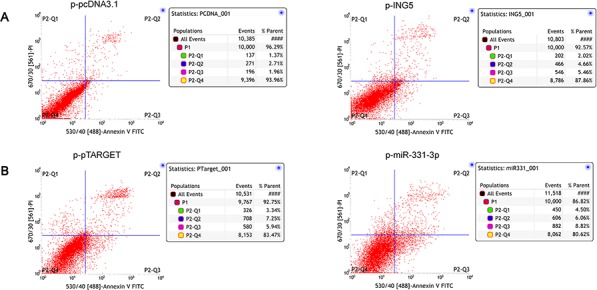
ING5 induces HCC cell apoptosis **A.** ING5 stable overexptession HCC cells (p-ING5) apoptosis were analyzed. p-pcDNA3.1 stable HCC cells were used as control. **B.** p-miR-331-3p, p-pTARGET (control) plasmids were transfected into ING5 stable overexptession HCC cells, apoptosis were analyzed by flow cytometry.

As the ING is known as an apoptosis inducer [20–22, 24], whether miR-331-3p/INR5 could inhibit apoptosis in the HCC? Cell apoptosis analysis was performed. We found that HCC cells showed enhanced apoptosis rates when ING5 was overexpressed compared to its control group (Figure 6A). And apoptosis rate was recovered when miR-331-3p was overexpressed in ING5 overexpression cells (Figure 6B). These results indicate that miR-331-3p/INR5 could inhibit apoptosis in the HCC.

### miR-331-3p or ING5 over-expression influence tumor growth in nude mice

**Figure 7:**
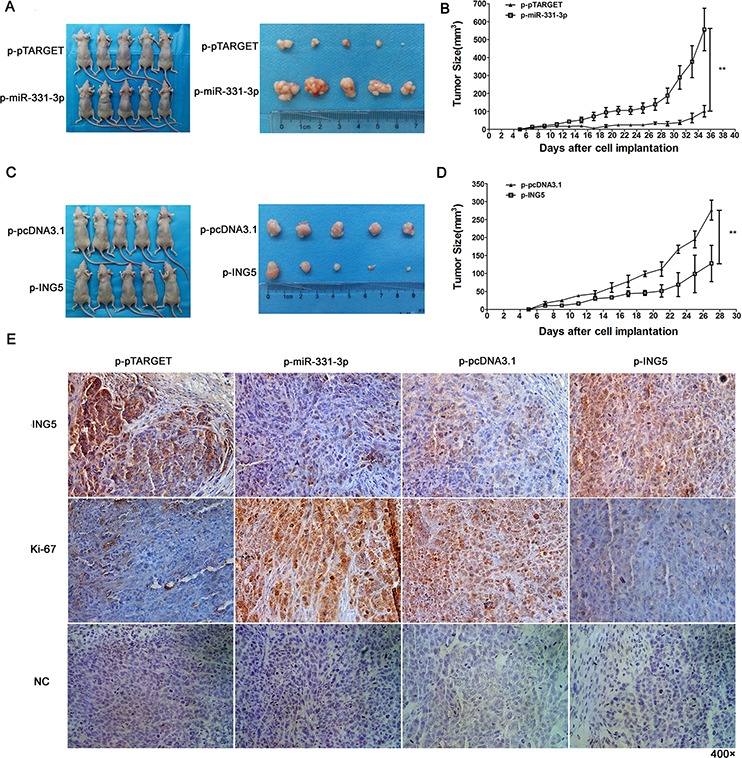
miR-331-3p or ING5 overexpression influences tumor growth in nude mice **A.** Representative pictures of tumors formed in p-pTARGET and p-pTARGET-miR-331-3p injected nude mice. **B.** Tumor growth curve of each group in A. **C.** Representative pictures of tumors formed in p-pcDNA3.1 and p-ING5 injected nude mice. **D.** Tumor growth curve of each group in C. *P < 0.05, **P < 0.01. **E.** Ki-67 and ING5 stained sections of transplanted tumors. Original magnification: 400×.

p-pTARGET, p-miR-331-3p, p-pcDNA3.1 or p-ING5 cells were injected subcutaneously into the upper left dorsal flank of nude mice. At 5 weeks after inoculation, mice injected with p-miR-331-3p cells had larger tumors compared to p-pTARGET (Figure 7A). Tumor growth curves showed that tumors grew faster in the p-miR-331-3p injected group (Figure 7B). At 4 weeks after inoculation, mice injected with p-ING5 cells had smaller tumors (Figure 7C) and these grew more slowly (Figure 7D).

To better understand the molecular mechanism of miR-331-3p and ING5 on tumorigenesis *in vivo*, Ki-67 and ING5 expression was measured in tumor tissues via immunohistochemistry. ING5-staining decreased in the p-miR-331-3p group and increased in the p-ING5 group compared with control. Also, staining intensity and the number of Ki-67 positive tumor cells increased in the p-miR-331-3p group and decreased in the p-ING5 group compared with their corresponding controls (Figure 7E). Therefore, miR-331-3p promoted tumorigenesis of hepatoma cells *in vivo* by regulating ING5 expression.

### ING5 was downregulated in human HCC tissue with HBV infection

**Figure 8:**
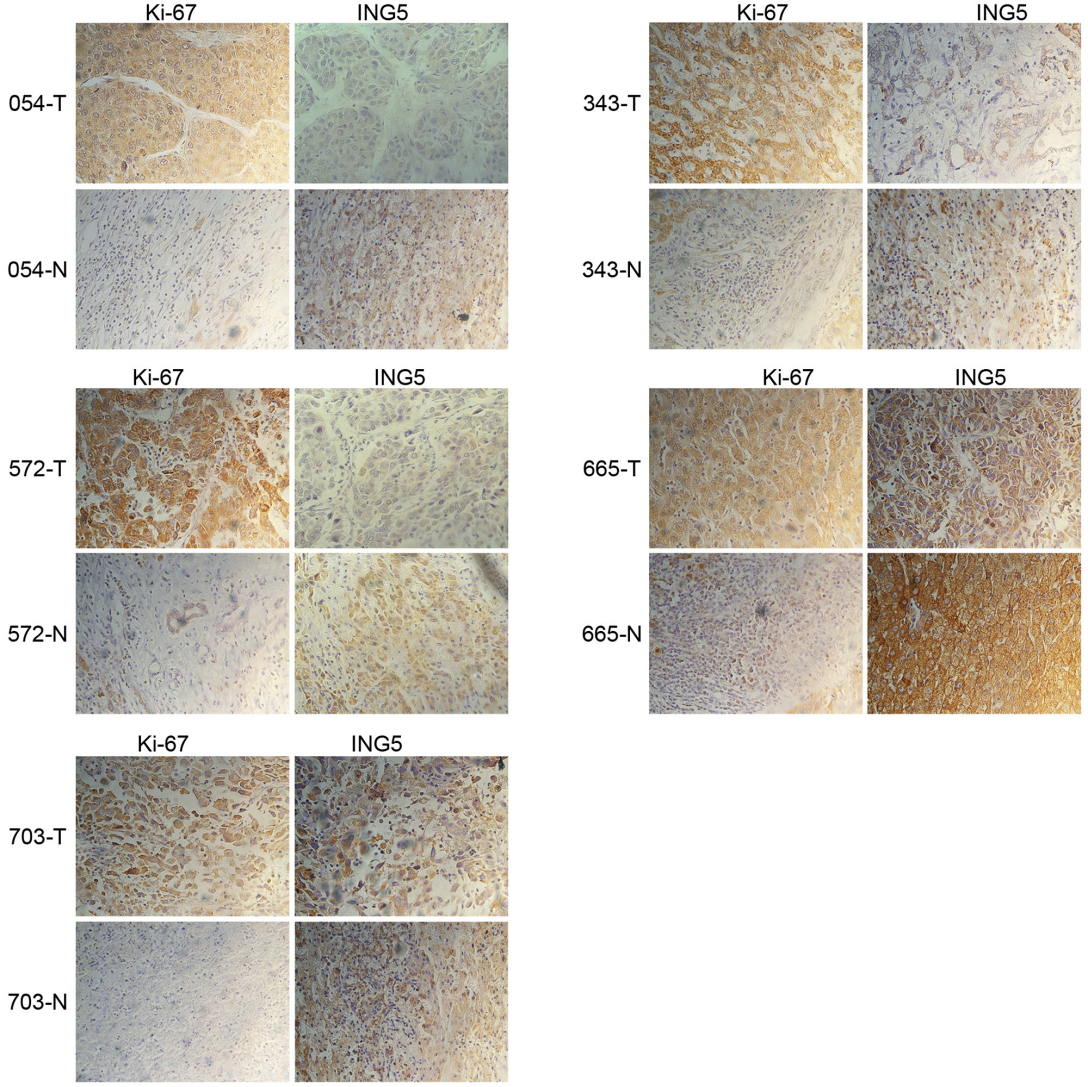
ING5 is downregulated in HBV-infected HCC tissue Human HBV-infected HCC tumor and pericarcinous tissue sections were immunohistochemistry staining with Ki-67 and ING5 (magnification 400×).

Because miR-331-3p was reported to be upregulated in human HCC tissue [15], we asked whether ING5 expression was correlated with miR-331-3p in HBV-HCC tissue. Immunohistochemistry revealed that ING5 expression was decreased in HCC tissue compared to corresponding pericarcinous tissue (Figure 8).

## DISCUSSION

It has been reported that 5% of the world's population (350 million people) is chronically infected with HBV, and locations with high incidence and mortality rates for HCC have high prevalence of chronic HBV infection [2]. miRNA dysregulation is known to be involved in human cancers, including HCC [25–28]; therefore, investigation of the association between abnormal expression of miRNAs and HBV in HCC may lead to new strategies for HCC prevention and therapy.

Here, we present evidence that miR-331-3p is upregulated in HCC cells lines that differentially express HBV. We also studied associations between HBV and miR-331-3p and showed that HBV increases miR-331-3p expression by enhancing its promoter activity. Furthermore, over-expression of miR-331-3p promoted proliferation of HCC cells both *in vitro* and *in vivo*.

Various studies showed that the dysregulated of miR-331-3p expression was associated with a variety of human cancers including prostate cancer, glioblastoma multiforme and gastric cancer and played a role as a tumor suppressor [29–31]. However, a recently study demonstrated that miR-331-3p promoted proliferation and metastasis of HCC [15]. In this report, we show that miR-331-3p expression was stimulated by HBV, which in turn inhibited the expression of a tumor suppressor gene ING5 and promoted proliferation of HCC. Our data suggest that miR-331-3p might act as an oncogenic factor in HCC cells. miR-331-3p may play different roles in different tissues and different types of cancers, so do other miRNAs. It has been reported for another miRNA called miR-143. miR-143 was dramatically upregulated in HBV-related HCC and promoted cancer cell invasion, migration and tumor metastasis by repression of FNDC3B expression [26], but miR-143 also repressed glioma cell migration, invasion, tube formation and slowed tumor growth and angiogenesis in a manner associated with N-RAS downregulation [32]. miR-17–5p, another well-known miRNA, has been reported to act as both an oncogene and a tumor suppressor in different cellular contexts by targeting different genes [33–35]. Thus, miRNAs have complicated functions in human cancer and these warrant investigation.

ING5 was reported to be a tumor suppressor gene that inhibited cell growth and induced apoptosis in certain cancers [20–22], but the effects of ING5 in HCC are poorly understood. In our research, we confirmed for the first time that ING5 was a target gene of miR-331-3p, and that miR-331-3p overexpression inhibited ING5. When miR-331-3p was suppressed, ING5 expression increased. ING5 expression was also inhibited by HBV, overexpression of ING5 inhibited cell growth and induced apoptosis of HCC cells. Thus, the proliferation effect of miR-331-3p depends on ING5 inhibition. As shown in Figure 5E, in pTARGET-miR-331-3p and pcDNA3.1-ING5 co-transfected groups, ING5 expression was greater than negative controls, but MTS assay indicated that cell proliferation was the greatest among the three. Thus, miR-331-3p-induced proliferation not only depends on suppression of ING5, but may require inhibition of other target mRNAs or signaling pathways. miRNA and its target mRNAs often form complex regulatory networks. Our data suggest that HBV upregulates miR-331-3p expression, which in turn inhibits multiple target genes that may be involved in HBV-associated HCC.

After confirming the HBV-miR-331-3p-ING5 interactions, we studied clinical diagnostic and therapeutic values of miR-331-3p and ING5. Chang and colleagues documented that [15] miR-331-3p was significantly upregulated (more than 2-fold; i.e., log_2_ [fold change] > 1) in 79 of 120 HCC cases. We measured ING5 expression in HCC tumor tissue derived from HBV infections by western blot and immunohistochemistry, and found that ING5 expression was decreased in human tumour samples compared to corresponding pericarcinous tissue. So, although we know more about HBV-related HCC, treatment is still problematic. We identified a link among HBV, miR-331-3p, and ING5 that is a novel constituent of HCC and this may offer a novel therapeutic strategy for HCC and may better define mechanisms underlying HCC on how it develops from HBV infection.

## MATERIALS AND METHODS

### Cell culture and transfection

All the cells lines used for this study were routinely used and preserved in our lab, including human hepatoma cell lines HepG2, HepG2.2.15, HepAD38 [36] and SMMC7721. HepG2, HepG2.2.15, and HepAD38 cell lines were cultured in minimum essential media (MEM) (Hyclone, China) supplemented with 10% fetal bovine serum (FBS) (Life Technologies, Grand Island, NY), 100 units/ml penicillin and 100 μg/ml streptomycin (Hyclone) and 1.2% sodium pyruvate (Hyclone). SMMC7721 cells were cultured in RPMI-1640 medium (Hyclone) supplemented with 10% FBS (Life Technologies) and 100 units/mL penicillin and 100 μg/ml streptomycin. HepG2, HepG2.2.15, HepAD38, and SMMC7721 cells were all maintained in a humidified incubator at 37°C with 5% CO_2_. Transfections were performed with Lipofectamine 2000 (Life Technologies) according to the manufacturer's instructions.

### RNA extraction and qRT-PCR

Total RNA from cell lines containing miRNA was isolated with Trizol reagent (Life Technologies) and treated with DNase (Promega, Madison, WI). First-strand cDNA was generated with a miRNA cDNA Kit or a BioRT cDNA First Strand Synthesis Kit according to the manufacturer's instructions (CWBio, Hangzhou Bioer Technology, China). Quantitative real time polymerase chain reaction (qRT-PCR) was performed using miRNA Real-Time PCR Assay Kit or UltraSYBR mixture (CWBio) to confirm expression of miRNAs or mRNAs. A cycle threshold (CT) was assigned at the beginning of the logarithmic phase of PCR amplification, and duplicate CT values were analyzed using the 2^−ΔΔCT^ method [37]. U6 and β-actin mRNA were used for normalization. The forward and reverse primers of qRT-PCR are given in Table 1.

**Table 1:** Primer sequences used for PCR or constructions of various plasmids

Amplifier primers	Primer sequence (5′-3′)
**Real-time PCR primer**	
miR-331-3p-F	GCCCCTGGGCCTATCCTAGAA
U6-F	AGAGCCTGTGGTGTCCG
U6-R	CATCTTCAAAGCACTTCCC
β-actin-F	GTGGATCAGCAAGCAGGAGT
β-actin-R	TGTGTGGACTTGGGAGAGGA
ING5-F	ACCAGAGGACGGAAGATAAG
ING5-R	TGCACTTGCTGTAGGCGTTC
VHL-F	ACATCGTCAGGTCGCTCTAC
VHL-R	ATCTCCCATCCGTTGATGTG
AKAP11-F	AGCTGGAGCAGTCTTGGTTT
AKAP11-R	ACTTCGTCCTCATGCTCTTC
ERBB2-F	ACCTGCTGAACTGGTGTATG
ERBB2-R	TGACATGGTTGGGACTCTTG
RGS-F	TGCCCATCAGAACAGTCAAG
RGS-R	ACTGGGTCCTCAATGGAAAG
DOHH-F	AGGCCTTCGATGACGATTC
DOHH-R	TGTCTTGCAGCACGTCCAC
SPOP-F	AGAGTCAACGGGCATATAGG
SPOP-R	AGCTTGTCATCAGGGAGAAG
TRERF1-F	ATGACCTCCAGCAAAGAGTG
TRERF1-R	TTGGCCTCAAACAGAGAGTG
**miR-331-3p over-expression primer**	
pTARGET-miR-331-3p-F	CGCCGCTCGAGATAATATCCTAAACAAAGCA
pTARGET-miR-331-3p-R	ACGCGTCGACTTTTAGGGCTAAGTTGCTTC
**miR-331-3p promoter primer**	
pGL3-Basic-miR-331-3p-F	CGGGGTACCAACCGCCTGACCAACATGGAG
pGL3-Basic-miR-331-3p-R	CCGCTCGAGTCCCTGGGACCATACCTAGAAC
**ING5 3′UTR primer**	
pGL3-Control-ING5-WT-F	AGCTCTAGAAGACCCATCTCTTGGATTG
pGL3-Control-ING5-WT-R	AGCTCTAGATCCAACACAATCTCTCCTG
pGL3-Control-ING5-MUT-F	TGAGAGGAATTTTAAGAAGCCCCAAGTGTAAG
pGL3-Control-ING5-MUT-R	CTTACACTTGGGGCTTCTTAAAATTCCTCTCA
**ING5 over-expression primer**	
pcDNA3.1-ING5-F	CGGGGTACCGCCACCATGGCGACCGCCATGTAC TTGG
pcDNA3.1-ING5-R	CCGCTCGAGCTACTTCTTCTTCCTCTTTTCC

### HBV assay

HBV surface antigen (HBsAg) or HBe antigen (HBeAg) expression was determined by ELISA using the conditioned medium. Conditioned medium from each cell line was assayed according to the supplier's recommendations (Diagnostic kit for Hepatitis B Virus surface antigen ELISA, diagnostic kit for Hepatitis B e antigen ELISA, Shanghai KeHua B-engineering, China) 48 h after transfection. Absorbance at 450 nm was measured using a Synergy HT Multi-Detection Microplate Reader (BioTek). Each cell line was tested in three independent experiments.

### miRNA target predictions

Predictions of miR-331-3p targets was performed using databases including miRbase (http://mirbase.org/), miRDB (http://mirdb.org/miRDB/, ref), http://microRNA.org-Targets and Expression (http://www.microrna.org/), and TargetScan (http://www.targetscan.org/).

### Plasmid construction

All sequences of cloning primers are illustrated in Table 1. The HBV expression plasmid pCH9/3091, constructed by Nassal's group (Heidelberg University, Germany), was a gift from Dr. Lan Lin (Southwest Hospital affiliated with the Third Military Medical University, China). A fragment of the miR-331-3p promoter region, designed using Eukaryotic Promoter Database (http://epd.vital-it.ch/), was amplified by PCR using the genomic DNA of HepG2.2.15 cells as the template. The amplified fragment was cloned into the *Kpn* I/*Xho* I sites of the pGL3-Basic vector (Promega) immediately upstream of the luciferase gene to generate pGL3-Basic-miR-331-3p. The miR-331-3p expression vector (pTARGET-miR-331-3p), a fragment encompassing the mature miR-331-3p sequence and its 5′-and 3′-flanking regions (694 bp in total) was amplified and then cloned into pTARGET™ Vector (Promega). The ING5 3′-UTRs fragment containing miR-331-3p binding site (CCAGGGG) was amplified by PCR from genomic DNA of HepG2.2.15 cells, and cloned into the *Xba* I site of pGL3-Control dual-luciferase miRNA target expression vector (Promega) immediately downstream of the luciferase gene. pGL3-Control-ING5-MUT, which carried the mutated 3′-UTR sequence (AAGCCCC) was generated based on pGL3-Control-ING5-WT plasmid by site-specific mutagenesis. The ING5 expression vector (pcDNA3.1-ING5) was created by cloning the ING5 coding sequence into the *Kpn* I/*Xho* I site of pcDNA3.1 (Life Technologies). All constructed vectors were confirmed by DNA sequencing.

### RNA interference

MiR-331-3p inhibitor, siING5 and its negative control (NC) were designed and synthesized by Invitrogen. Sequences were as follows, miR-331-3p inhibitor: 5′-UUCUAGGAUAGGCCCAGGGGC-3′, random microRNA inhibitor NC: 5′-CAGUACU UUUGUGUAGUACAA-3′, siING5: 5′-UUUCUUAUCU UCCGUCCUCUG-3′, siHBx: GUUUAAGGACUGGG AGGAGC, random scramble siRNA NC: 5-CGCG AAUACGGAAAAGGAAUG-3.

### Luciferase reporter assay

For the luciferase reporter assay, SMMC7721 cells were seeded in a 24-well plate at a density of 45% confluent and co-transfected with 250 ng pTARGET-miR-331-3p or pTARGET vector, 150 ng of pGL3-Control-ING5-WT or pGL3-Control-ING5-MUT and 25ng pRL-TK plasmid expressing *Renilla* luciferase (Promega). HepG2.2.15 cells were seeded in 24 well plates at a density of 60% confluent and co-transfected with 70 pmol miR-331-3p inhibitor or inhibitor NC, 150 ng of pGL3-Control-ING5-WT and 50 ng pRL-TK. Cells were collected 48 h after transfection and analyzed using the Dual-Luciferase Reporter Assay System (Promega, Madison, WI). Relative luciferase activity was normalized to *Renilla* luciferase activity. Transfections were performed in triplicates and repeated at least 3 times in independent experiments.

### Western blot

Cells were lysed with 1% RIPA Lysis Buffer (Beyotime, China) 48 h after transfection. Supernatants were collected, and protein was measured using the BCA Assay Kit (Beyotime). Protein samples were separated by 10% SDS-PAGE and then transferred to a PVDF membrane. The membrane was blocked with 5% milk, incubated overnight at 4°C with a primary rabbit antibody against human ING5 (Proteintech, China, 1:1000 dilution), washed three times in TBST and then incubated with a goat anti-rabbit HRP secondary antibody (Bioword, USA, 1:5000 dilution). Finally, bound antibody was detected using an ECL Detection Reagent (Millipore, Billerica, MA). Data were normalized to β-actin (Bioword, 1:5000 dilution).

### Stable cell line generation

SMMC7721 cells were transfected with pTARGET, pTARGET-miR-331-3p, pcDNA3.1 or pcDNA3.1-ING5 and selected with G418 (1,000 g/ml). Two weeks later, few cells survived, and G418 was reduced to 500 μg/ml. Stable cell lines p-miR-331-3p and p-ING5, which could stably express miR-331-3p or ING5 were established and expression was measured via Real time RCR and Western blot, respectively.

### MTS assay

SMMC7721 cells were trypsinized and seeded into 96-well culture plates 24 h after transfected specific DNAs or RNAs as indicated in result (4,000 cells/well). Cells were harvested at different time points (24, 48, 72, and 96 h) for cell proliferation assay using an MTS kit (cellTiter96AQ, Promega, Madison, WI) following the manufacturer's protocol and the absorption was read at 490 nm.

### Colony formation assay

Twenty-four hours after transfection, cells were trypsinized and seeded into 6-well plates with a density of 2,000 per well. When cells were visible colonies (about 1 week), colonies were washed with PBS three times. Cell colonies were fixed and stained with a crystal violet cell colony staining kit (GenMed Scientifics, city, state) according to the manufacturer's instructions. Colonies were counted under a microscope. Clone formation rate was calculated according to the formula: Clone formation rate = clone forming number/Inoculation cell number × 100%.

### Tumor transplantation

Female BALB/c nude mice (4–6 weeks-of-age) were purchased from the Laboratory Animal Services Center of Chongqing Medical University. Animal handling and experimental procedures were approved by the Animal Experimental Ethics Committee of Chongqing Medical University. A total of 3 × 10^6^ p-pTARGET or p-miR-331-3p cells, 5 × 10^6^ p-pcDNA3.1 or p-ING5 cells were injected subcutaneously into the upper left dorsal flank of nude mice (*N* = 5). Tumor volumes were measured every 2 days. Four (p-pcDNA3.1 and p-ING5) or five (p-pTARGET and p-miR-331-3p) weeks later, mice were euthanized and tumors were dissected. Tumor volumes were calculated using the equation V (mm^3^) = *A* × *B* ^2^/2, where A is the largest diameter, and B is the perpendicular diameter.

### Apoptosis assay

Cells in 6-well plates were transfected for 24 h followed by serum deprivation for another 48 h. The cells were then harvested by trypsinization, washed with PBS, and resuspended in 1 ml PBS. Apoptosis in HCC cells was quantified by staining with fluorescein isothiocyanate (FITC)-Annexin V and PI. The stained cells were immediately analyzed by flow cytometry.

### Immunohistochemistry

Paraformaldehyde-fixed, paraffin-embedded tissues of transplanted tumors were sectioned at 4.5 μm thickness and Ki-67 and ING5 expression were measured (Bioword, 1:50 dilution; Proteintech, 1:80 dilution, respectively). Visualization was achieved using 3,3′-diaminobenzidine substrate and sections stained with no primary antibodies were negative staining controls. The staining images were collected using a microscope (Carl Zeiss, Germany) and at a original magnification of 400×.

### Patient samples

HBV-HCC tissues and paired pericarcinous tissues were obtained from patients who underwent surgery for HCC at the 1st or 2nd Affiliated Hospitals of Chongqing Medical University between 2010 and 2012, with the approval of the Institutional Ethical Review Board of Chongqing Medical University. Patients offered informed consent and had no history of chemotherapy or radiation therapy before surgery. All liver specimens were immediately collected after surgery and stored at −80°C until further use.

### Statistical analysis

Data are expressed as means ± standard deviation. Statistical analysis was performed with an independent *t*-test. *p* < 0.05 was considered statistically significant.
